# Proto-sulphuret of Iron, a New Antidote for Corrosive Sublimate

**Published:** 1843-03

**Authors:** 


*Proto-sulphuret of Iron, a new Antidote for Corrosive Sublimate.*—By Mialhe.—It results from my experiments, that the protosulphuret of iron, a totally inert article, instantly decomposes corrosive sublimate, giving rise to two inoffensive compounds—proto-chloride of iron and deutosulphuret of mercury. This invaluable property leads me to announce the protosulphuret of iron, in the form of hydrate, as affording by far the best antidote for this poison.

At some future time I will publish the details of my chemical researches, as well as the results of the physiological experiments which I propose to institute on this subject. In the mean time, I advance a chemico-physiological proof in favor of the efficacy of this antidote which appears to possess real value.

Whenever a few centigrammes of corrosive sublimate is placed in the mouth, it immediately produces its characteristic insupportable metallic taste. It is then sufficient to wash out the mouth with the hydrated protosulphuret of iron, in the state of a thin pulp, a condition in which it should always be used, to cause all the metallic taste to disappear as if by enchantment. This fact needs no commentary. It speaks for itself, without need of any explanation.

This antidote is not restricted in its effects to the soluble compounds of mercury—it serves also to destroy the injurious action of many other metallic salts, and particularly those of copper and lead.

To prepare the protosulphuret of iron, any quantity of pure protosulphate of iron is to be dissolved in at least twenty-four times its weight of distilled water, which has been boiled to drive off any atmospheric air; this solution is to be precipitated by a sufficient quantity of protosulphuret of sodium, likewise dissolved in boiled distilled water. The protosulphuret of iron thus formed is to be washed with pure water, and preserved for use in a closely stopped bottle, which is to be completely filled with distilled water.

Although the protosulphuret of iron may be made in a few moments, it is nevertheless proper that it should be kept ready prepared, to avoid the loss of any precious moments in a case of poisoning.

The direction to preserve this sulphuret from contact of the air should be very strictly followed, as this compound has a strong tendency to pass to the state of sulphate.—*American Jour. of Pharm.*, from *Journal de Pharm. and Chim.*

